# A Case of Infantile Scurvy or Scurvy-Rickets

**Published:** 1903-12

**Authors:** Christopher Elliott

**Affiliations:** Senior Physician to the Royal Hospital for Sick Children and Women, Bristol.


### A CASE OF INFANTILE SCURVY OR SCURVY-RICKETS.

BY

Christopher Elliott, B.A., M.D.,

*Senior Physician to the Royal Hospital for Sick Children and Women, Bristol.*

Cases of the above kind are by no means common, and I think the following is for this reason alone worthy of being put on record.

In February, 1902, I was asked by Dr. McQuade of this city to see an infant aged eight months. It appeared that for the past six weeks the child would scream whenever she was moved or touched. At first she was thought to be suffering from rheumatism, but about ten days ago both thighs were found to be greatly swollen, especially the right. The mother stated that this swelling had come on suddenly one night. The thighs felt hard and indurated, but did not crackle or pit upon pressure. The left measured nine inches in circumference, and the right ten inches. The legs and upper extremities were not affected. There was no craniotabes, the ends of the long bones were not enlarged, and there was no beading of the ribs. The child looked very ill and anæmic. There were no ecchymoses beneath the skin or into the conjunctiva, neither had there been any bleeding from the nose, gums, stomach, bowels, or kidneys. Two lower incisor teeth had been cut, and there were signs of the corresponding two upper being about to come through. In reply to my enquiries, the mother informed me that she had been unable to suckle her infant, and as cow's milk had always caused sickness when it was given, she had rigidly excluded it from her baby's diet, and had brought it up entirely on a proprietary food. I pointed out to her the mistake she had made, and prescribed a change of diet and the daily administration of some fresh lemon juice, and under this treatment the little patient made a slow but complete recovery.

*Remarks.*—It is only during recent years that the attention of the profession has been drawn to this class of cases. Cheadle was the first in this country to do this, and was closely followed by Barlow, who was able to throw more light on the subject, as he had the opportunity of making a *post-mortem* in two out of eleven cases which he had seen.

That the disease is caused by the too prolonged use of one kind of food, such as sterilised milk or a proprietary food, is established beyond doubt by a record of cases by careful observers, and the rapid improvement which often follows upon a change of diet. The name "Scurvy-Rickets" should, I think, be now abandoned, for the disease is scorbutic more than diathetic; the association of rickets is rather accidental, and owing to the age at which the disease is usually met with, namely, from six to fifteen months.

Tornp, of Christiania, has advanced the view that scurvy is due to ptomaine poisoning, but I do not think that this theory will satisfactorily explain the changes met with in infantile cases, and in what way they are produced is still a matter for further investigation. Shortly before seeing Dr. McQuade's patient I had the opportunity of being present at a *post-mortem* in a fatal case. In this infant the lower extremities were also affected, and there was not only sub-periosteal hemorrhage, but there was infiltration of blood into the cancellous tissue of the long bones, as well as into the soft parts surrounding them.

